# High genetic variation, low differentiation, and Pleistocene expansions of the migratory and endangered long-nosed tequila bat, *Leptonycteris nivalis*, inferred using both maternal and paternal genetic markers

**DOI:** 10.1371/journal.pone.0316530

**Published:** 2025-01-09

**Authors:** Roberto-Emiliano Trejo-Salazar, Jaime Gasca-Pineda, Katia Hernández-Bolaños, Dulce-Carolina Hernández-Rosales, Rosalinda Tapia-López, Erika Aguirre-Planter, Rodrigo A. Medellín, Livia León-Paniagua, Luis E. Eguiarte

**Affiliations:** 1 Facultad de Ciencias, Departamento de Biología Evolutiva, Universidad Nacional Autónoma de México, Ciudad Universitaria, Mexico City, México; 2 Departamento de Ecología Evolutiva, Instituto de Ecología, Universidad Nacional Autónoma de México, Mexico City, Ciudad de México, México; 3 Departamento de Conservación de la Biodiversidad, El Colegio de la Frontera Sur, Villahermosa, Tabasco, México; 4 Departamento de Ecología de la Biodiversidad, Instituto de Ecología, Universidad Nacional Autónoma de México, Mexico City, Ciudad de México, México; National Cheng Kung University, TAIWAN

## Abstract

Tequila bats (genus *Leptonycteris*) have gained attention for their critical role in pollinating different plant species, especially *Agave* spp. and columnar cacti. *Leptonycteris nivalis* is the largest nectar-feeding bat in the Americas, and the females exhibit migratory behavior during the breeding season. Due to its relatively small and seemingly declining population sizes, this species is protected by government agencies in the United States and Mexico. We conducted population genetics and phylogeographic analyses to elucidate the genetic structure and demographic history of the species using two mitochondrial markers and a Y chromosome-associated gene, to describe both maternal and paternal lineages. We estimated high haplotypic diversity measures for the different markers (*Dloop*—Hd = 0.775; *Cyt-b*—Hd = 0.937; *DBY* -Hd = 0.946). We found that geographic genetic differentiation is very low, and there is high connectivity among localities. The estimated divergence time between *L*. *nivalis* and *L*. *yerbabuenae*, the other species in the genus found in Mexico, aligns with previous estimates for the genus (6.91–9.43 mya). A demographic expansion was detected approximately at 600 ka—700 ka (thousands of years ago). The historical demographic changes observed in *L*. *nivalis* appear to be associated with environmental shifts during the Pleistocene, which likely impacted the distribution range of the plants that these bats feed on, such as *Agave* species.

## Introduction

Bats are one of the most remarkable groups of mammals worldwide due to their nocturnal behavior, flight ability, unique physical characteristics, complex immune system, high metabolism, vast diversity, and the ecosystem services they provide [1]. Nectar-feeding bats, in particular, are important pollinators for many plants, especially those in the families Asparagaceae, Cactaceae, Malvaceae, Fabaceae, Bignonaceae, and Convolvulaceae. Among these, there are economically valuable species such as agaves (family Asparagaceae), which are used in the production of mezcal, tequila, and other distilled beverages in Mexico. Consequently, nectar-feeding bats have received increased attention in conservation strategies [2–4].

The two best known nectar-feeding bats of North America (including all of Mexico) are *L*. *nivalis* and *L*. *yerbabuenae*, recognized for their behavior, ecology, and abundance. These bats visit numerous flowers every night and can travel long distances, making them highly efficient pollinators, especially for many agave and cactus species. They have developed strong ecological relationships with these plants [5,6], and *Leptonycteris* species, particularly *L*. *yerbabuenae*, can form large colonies of up to tens of thousands of individuals (especially *L*. *yerbabuenae*), consuming significant amounts of nectar to meet their energy needs [7].

The Mexican long-nosed bat, *Leptonycteris nivalis*, is the largest nectar-feeding bat species in North America. This species is distributed from southern Texas in the United States through central Mexico, reaching as far south as the states of Morelos, Oaxaca and Guerrero [5,8].

Conservation efforts have been undertaken in both the United States and Mexico [9]. The Mexican long-nosed bat is classified as *Endangered* by the US Fish and Wildlife Service, and as *Threatened* by the Mexican environmental agency’s NOM-059 list. Additionally, it is listed as an *Endangered* species on RedList of IUCN (EN-A2c, ver. 3.1; [10]) due to its relatively low and apparently declining populations, which are likely a result of habitat fragmentation and perturbation [5].

Mexican long-nosed bats exhibit a distinctive migratory behavior, with some females flying north to maternity caves during the spring and summer seasons [11,12]. During this period, some reproductive females migrate from the southern to the northern part of the species’ range. One of the largest maternity refuges for *L*. *nivalis* is located in Emory Cave, Texas. In autumn and winter, these females return south along with their offspring. However, several aspects of their migratory behavior are still unknown. For example, it is unclear whether *L*. *nivalis* roosts are genetically connected, or if they form isolated philopatric colonies [13]. Additionally, it is not known, where non-migrant males take refuge while the females migrate.

Previous studies suggest that the timing of migration is linked to the blooming periods of the different agave species in the northern part of their range during summer [14,15]. However, the migration process is complex, as in the southern part of their distribution all males and some females remain in roosting caves year-round [11,16]. This raises questions about the proportion of the female population that migrates.

Annual migration is a critical aspect of the natural history of species for ecological and evolutionary history analyses and for its conservation. In particular, migration can affect genetic structure, especially in cases where each sex exhibits different migration patterns during the reproductive season [17,18]. Differences in allelic frequencies may be expected between locations where the species is present year-round and those with migratory populations. This is due to a potential reduction in gene flow among localities or populations in the non-migratory male populations. Similar patterns have been observed in other volant species, such as birds and other bats, where populations show genetic structure due to lack of seasonal movement [17,19].

Migration, habitat fragmentation, and disturbance, as well as different environmental conditions, can significantly impact *L*. *nivalis*. Habitat fragmentation, as documented [5], may lead to genetic isolation of populations, resulting in a decline in genetic diversity [20] due to inbreeding, genetic drift and/or natural selection [21]. However, *L*. *nivalis* appears to have undergone a historical geographic expansion, likely correlated with the spread of succulent plants of the genus *Agave*, and other food sources, particularly columnar cacti (Cactaceae) [22,23] which are now dominant plants in the arid and semi-arid regions of Mexico and southern USA.

*Leptonycteris nivalis* has received comparatively less attention than its close relative *L*. *yerbabuenae*. This is partly due to the smaller population size and more restricted distribution of *L*. *nivalis*, which has resulted in limited knowledge about its roosting sites and migratory movements. Despite this, there is now sufficient information on both species to enable an initial comparison of their ecological characteristics, including migratory behavior and feeding habits. However, obtaining molecular data remains crucial for inferring potential genetic differentiation patterns, as well as understanding demographic historical dynamics, which would allow for a more comprehensive comparison between the two *Leptonycteris* species.

Several geological and climatic changes in the recent past (Pleistocene) have influenced speciation, extinction and diversification in the Mexican flora [24–26] and fauna [27,28]. These patterns have been examined using both biogeographic and phylogeographic strategies [26,27]. In particular, there are two recent genetic studies on *L*. *nivalis*. Ammerman et al. (2019) analyzed 72 bats from 8 localities using a nuclear (bi-parental) dominant molecular marker (*AFLP*, Amplified Fragment Lengths Polymorphism), and a mitochondrial (maternally inherited) marker (*D-loop*) [29]. Pourshoushtari and Ammerman (2021) [12] analyzed seven nuclear microsatellite loci and included 113 bats from two distant localities across the species’ entire distribution. Both studies concluded that there were no significant genetic differences among localities, highlighting a strong genetic connectivity across populations. The more recent study [26] did not consider sex-specific markers to infer genetic structure, considering the migratory patterns of females or the more sedentary behavior of males [13].

In the present study, we analyzed paternally and maternally inherited molecular markers to disentangle the effects of sex bias in migration patterns and to understand the distribution of genetic diversity in *L*. *nivalis*. Our phylogeographic analysis included nine localities across the species distribution, encompassing 153 individuals from both sexes in Mexico and Texas, USA. The aim was to determine historical demography and genetic barriers among sites.

Historical distribution changes have been previously suggested for *L*. *nivalis* and its closest relative, *L*. *yerbabuenae*, both of which coincide with the expansion of the genus *Agave*, one of their primary food sources [30]. Given the current migratory movements of females, we anticipated little to no genetic differentiation among sampled localities and high genetic connectivity, especially for maternally inherited markers.

Thus, we hypothesized that *L*. *nivalis* would exhibit low geographic genetic differentiation and high connectivity among localities, as well as evidence of a historical demographic expansion. This expansion likely coincided with past global climate changes (global warming and cooling) during the Pleistocene, particularly the Last Interglacial and Last Maximum Glacial (130 kya and 20 kya respectively), periods marked by significant temperature fluctuations. These climatic conditions may have been favorable for the expansion of this species.

## Materials and methods

**Fig 1:**
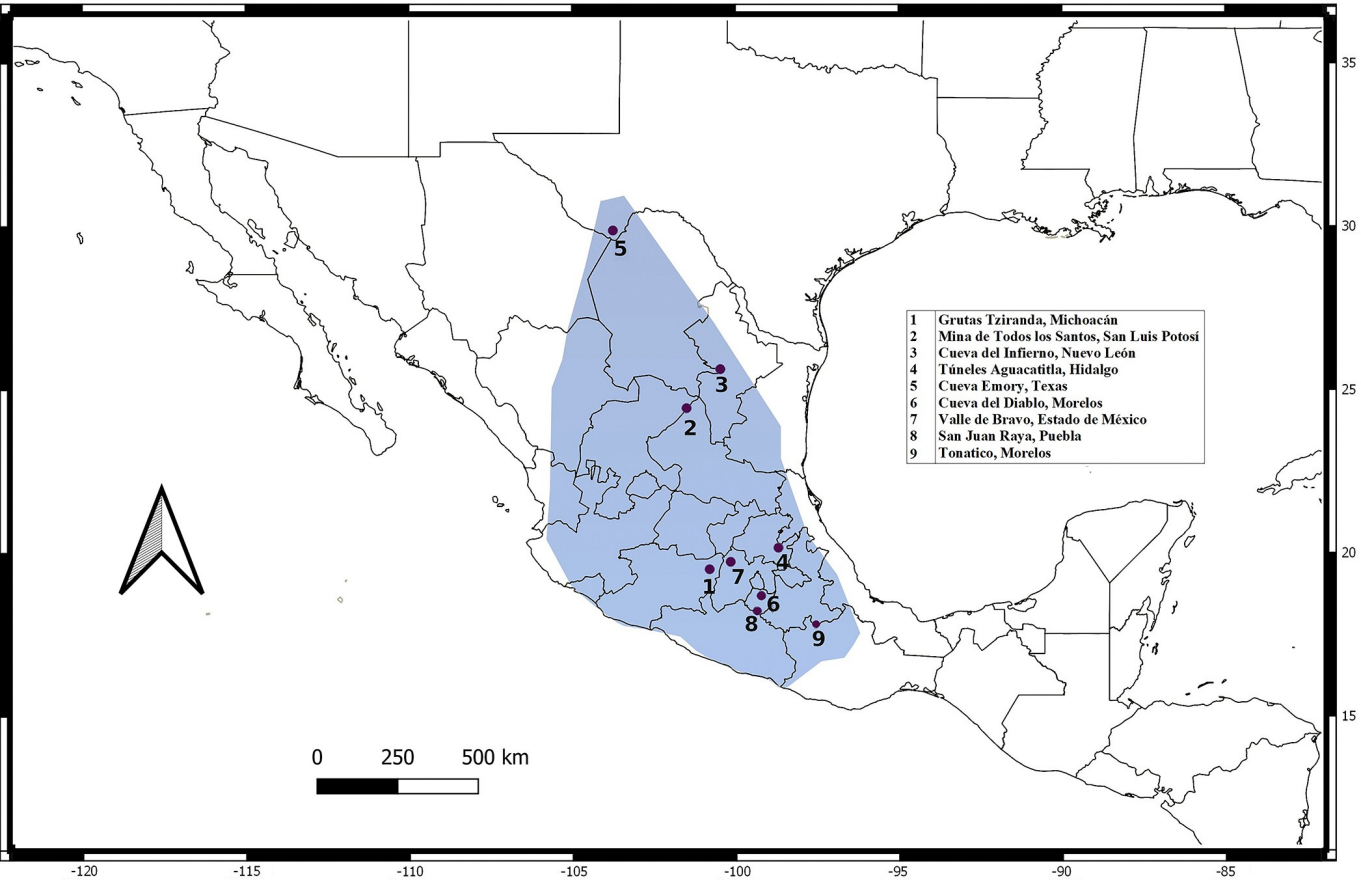
Distribution and sampling sites for *Leptonycteris nivalis*. Current distribution of *Leptonycteris nivalis* according to IUCN [10]. Pink dots represent our sampling localities and red numbers correspond to the locality’s designated number in Table 1. Basemap was taken from (CONABIO—http://geoportal.conabio.gob.mx/metadatos/doc/html/dest22gw.html).

Samples were taken from nine localities in roosting caves, and by using mist nets on field feeding-sites along *L*. *nivalis* distribution from 2014 to 2016 (Fig 1, Table 1). The samples were obtained with the scientific collecting permit “Secretaría del Medio Ambiente y Recursos Naturales (SEMARNAT) SGPA/DGVS/07161/15” following the Animal Care and Use protocols of the American Society of Mammalogists [31]. Tissue samples were taken with a 3 mm^2^ biopsy wing punch in an area of the wing with no blood capillaries or nerve terminals. Wing biopsies were fixed in 90% ethanol at environmental temperature and then stored at -20°C until DNA extraction.

**Table 1:** Number of male and female samples of *Leptonycteris nivalis* sequenced for mitochondrial *Cyt-b*, *D-loop*, and chromosome Y *DBY* regions (only males), for each sampled locality and state from Mexico and USA (locality ID key is shown in the column next to each locality) and Haplotype diversity (*Hd*) for each locality.

		*Cyt-b*				*D-loop*				*DBY*	
Locality		Male	Female	Total	*Hd*	Male	Female	Total	*Hd*	Total	*Hd*
**1 Grutas Tziranda, Michoacán**	Tzi	12	5	**17**	0.9044	10	4	**14**	0.8022	**11**	0.9273
**2 Mina de Todos los Santos, San Luis Potosí**	San	7	11	**18**	0.8105	8	11	**19**	0.8363	**8**	0.8333
**3 Cueva del Infierno, Nuevo León**	Inf	2	19	**21**	0.9714	1	16	**17**	0.5882	**2**	0
**4 Túneles Aguacatitla, Hidalgo**	Agu	5	6	**11**	0.9818	8	11	**19**	0.8363	**6**	0.8
**5 Cueva Emory, Texas**	Emo	23	34	**57**	0.9198	20	35	**55**	0.7650	**18**	0.9935
**6 Cueva del Diablo, Morelos**	Dia	12	3	**15**	0.9905	3	1	**4**	1	**12**	0.9091
**7 Valle de Bravo, Estado de México**	Peñ	0	0	**0**	-	0	0	**0**	-	**5**	1
**8 San Juan Raya, Puebla**	Juan	9	0	**9**	0.9722	0	0	**0**	-	**4**	1
**9 Tonatico, Morelos**	Ton	2	3	**5**	1	2	2	**4**	1	**2**	1
** **	** **			**153**				**132**		**69**	

We believe that our sampling is both adequate and representative of the species’ distribution, covering most of its known range and including individuals from their key refuges. Our sampling design accounted for the migratory patterns and seasons for *L*. *nivalis*. Nevertheless, this is challenging, as the specific roosting sites of non-migratory individuals are unknown, complicating the sampling process. Despite this, our approach allows us to infer potential genetic differences indirectly, based on haplotype distribution and the spatial segregation of males and some females, as well as the philopatry of migratory females [32]. Additionally, the use of maternally and paternally inherited molecular markers enables us to assess the genetic diversity of *L*. *nivalis*, which, despite differences between mitochondrial markers, is generally high for the species.

### DNA extraction and amplification

Total genomic DNA was extracted following Paboö’s modified protocol [33]. Tissue was digested for 12 h at 40°C in Paboö lysis solution (100mM NaCl, 100mM Tris HCl, and 2mM EDTA, pH8.0) with 20 mg/ml proteinase K, 2% SDS and 0.04M DTT, followed by a phenol: chloroform protocol for DNA isolation [33]. The quality and concentration of extracted DNA were visualized in a 1.0% agarose gel. We performed gel electrophoresis at 90 V for 30 minutes; gel was stained with Midori green advance solution and visualized in UV light.

We analyzed two mitochondrial DNA regions: cytochrome b (*Cyt-b*) and control region (*D-loop*), which are maternally inherited and variable in most mammal species [34]. We also analyzed the Dead-box region from the Y chromosome gene (*DBY*), which is paternally inherited; this marker has been used before for phylogeographic studies in phyllostomid bats showing adequate variation and resolution to infer phylogeographic patterns [35,36].

We amplified a fragment of 1121 bp of *Cyt-b* with primers L14125 5’TGAAAAAYCATCGTTGT 3’ and H15915 5’TCTTCATTTYWGGTTTACAAGAC 3’ [37]. For the *D-loop* region we amplified a fragment of 828 bp with primers L15933 5′-CTCTGGTCTTGTAAACCAAAAATG-3′ and H637 5′-AGGACCAAACCTTTGTGTTTATG-3′ [38]. PCR for mitochondrial markers was performed in a final total reaction volume of 15 μl, and contained 2μl of DNA, 2 U of Taq polymerase (GoTaq Flexi DNA Polymerasa, Promega, USA), 0.4 μM of each primer (10μM), 1x Taq buffer, 2.5 μM of MgCl₂ (25μM), 0.2 μM of dNTPs (10μM) and 7.325μl of H_2_O. The PCR profile for *Cyt-b* was: 5 min of initial denaturation at 95°C, followed by 35 cycles of 30 s at 96°C, 1 min at 53°C, 2 min at 72°C, and a final extension of 7 min at 72°C; and for *D-loop*: 3 min at 95°C, followed by 35 cycles of 30 s at 94°C, 45 s at 54°C, 2 min of 72°C, and a final extension of 10 min at 72°C in an ABI Veriti 96-Well Thermal Cycler (Model: 9902; Thermo Fisher Scientific Inc.).

For the Dead-box region from the Y chromosome gene (*DBY*), we amplified a fragment of 482 bp with primers 5’-CCGTTACTTCCATTTTCAAAA-3’ and 5’-GCTAAAACCAACGAGATTGGT-3’ [39,40]. The reaction mixture of 15μl total volume contained 2μl of genomic DNA, 2 μM of each primer (10μM), 200 μM dNTPs (10μM), 1.5 mM MgCl_2_ (25μM), 2.5 U of Taq DNA polymerase (Promega) and 7.7μl of H_2_O. Amplification was carried out as follows: 10 min of an initial denaturation at 94°C, 36 cycles of 45 s at 94°C, 30 s at 54°C, 2:30 min at 72°C, and a final extension of 5 min at 72°C in an ABI Veriti 96-Well Thermal Cycler (Model: 9902; Thermo Fisher Scientific Inc.). We sent our amplified products of each genetic region for forward and reverse sequencing to Macrogen USA’s Maryland headquarters (http://www.macrogenusa.com).

We attribute the variation in final sample numbers for the different markers to PCR amplification issues and artifacts. *Cyt-b* was successfully amplified in most of the individuals, while *D-loop* amplifications and sequences yielded lower quality in some cases. We believe this was due to PCR artifacts and the condition of samples during transportation, which likely caused DNA degradation. Additionally, some samples had limited tissue available, resulting in lower DNA quantities for analysis. Consequently, these samples were excluded from further analyses. A total of 154 individuals were amplified for *Cyt-b*, 138 for *D-loop* and 69 for *DBY* (Table 1).

All sequences are available in NCBI GenBank (accession number: *Dloop*: OQ971506—OQ971533; *Cytb*: OQ971411—OQ971505; *DBY*: OQ971534—OQ971577).

### Data analysis

#### Genetic diversity

We evaluated the quality of DNA sequences and then assembled forward and reverse sequences using Consed 29.0, with default settings [41,42]. Subsequently, we aligned the sequences using CLUSTAL X [43], and manually verified the alignment. Missing data or undetermined bases were excluded from analyses, and sequences with more than 50% of missing data were removed.

For each marker, we estimated the number of segregating sites (S), number of haplotypes (h), haplotype diversity (*H*_*d*_), and nucleotide diversity (π) for each sampled locality. These calculations were performed using DNAsp 5.10.01 [44] and Arlequin 3.5.1.2 [45]. For the evolutionary and historical demography analyses, we concatenated both mitochondrial markers to compare maternal versus paternal lineages (i.e., dated genealogies, distance genealogies, sky-line plots, and Monmonier’s analyses).

#### Population genetic structure

We performed an analysis of molecular variance (AMOVA) with 1000 permutations and a confidence level of 95%, including the overall fixation index statistics (*F*_ST_) with 10,000 permutations using Arlequin [45]. All samples were treated as a single group to determine the amount of variation partitioned among and within localities or groups [45].

We also performed a principal component analysis (PCA) using the R package ADEGENET version 2.0.1 to summarize genetic similarities among individuals and a dendrogram based on the agglomeration method, using complete linkage performed with command hclust using Euclidean distances (R package, ADEGENET [46]).

#### Connectivity

To identify barriers among sample sites, we employed the Monmonier algorithm [47] using ADEGENET in R. In this analysis, we used a Delaunay triangulation to establish a connection network among localities. The Monmonier’s algorithm detects boundaries among vertices of a valuated graph based on genetic distances. The localities’ scores obtained from the previously estimated PCA were employed as a distance matrix [47]. The distance threshold between immediate neighbors was determined based on an abrupt decrease between connected points, as suggested by the author [46]. We used the function optimize.monmonier to obtain the optimal boundaries.

### Historical analyses

#### Divergence times

To estimate divergence times, we first determined the substitution model that best fits our data using jModelTest 2 [48], based on the Akaike Information Criterion (AIC; Akaike, 1974). Thus, mitochondrial *Cyt-b* and *D-loop* sequences were combined into a concatenated data file, using the GTR+G+I model with γ-distributed rate heterogeneity. For chromosome Y *DBY* gene, we employed the GTR+G substitution model.

For each region, we generated an ultrametric tree and estimated divergence times under a relaxed uncorrelated lognormal clock model employing BEAST 1.10.4 [49], which allows rates to vary among branches. Outgroup sequences were obtained from GenBank for each region. The bat species used as outgroups were selected based on their close phylogenetic relationships with *L*. *nivalis*, based on previous research involving another species of the genus, *L*. *yerbabuenae* [36]. Due to data availability, different species were employed as outgroups for analysis of the different regions. For *Cyt-b* we used *Glossophaga morenoi* (GenBank accession number: AF382882.1), *Glossophaga soricina* (GenBank accession number: MN719369.1), *Leptonycteris yerbabuenae* (GenBank accession number: MH198749.1), *Leptonycteris curasoae* (GenBank accession number: AF382889.1) and *Pteronotus parnellii* (GenBank accession number: KX787994.1) as outgroups. For *D-loop*, the outgroups were *Glossophaga morenoi* (GenBank accession number: MF804238.1), *Glossophaga soricina* (GenBank accession number: MH156139.1), *Leptonycteris yerbabuenae* (GenBank accession number: MT790834.1), *Leptonycteris curasoae* (GenBank accession number: AF510563.1) and *Pteronotus parnellii* (GenBank accession number: U95322.1). Outgroups for the *DBY* genealogy were *Glossophaga soricina* (GenBank accession number: JF458413.1), *Uroderma bilobatum* (GenBank accession number: JF458602.1), *Platyrrhinus helleri* (GenBank accession number: JF458470.1), *Trachops cirrhosus* (GenBank accession number: JF458543.1) and *Pteronotus parnellii* (GenBank accession number: JF459336.1).

In all three cases, genealogies were calibrated using four dates. Two calibration points, derived from the fossil record: Glossophaginae 22.8 million years ago (mya) (1.5 Standard Deviation, SD) [50] and Choeonycterinii at 13 mya (1.0 SD) [51]. Calibration points for *Glossophaga* + *Leptonycteris* clade at 15 mya (1.0 SD) and *Leptonycteris* at 12 mya (1.0 SD) were derived from previously reported detailed Bayesian analyses [52].

Priors for BEAST 1.10.4 were set with default values, running for 500 million generations with sampling every 1000 generations, and a 10% burn in. Convergence and stationarity of 10,000 trees were assessed using Tracer 1.7.1 [53]. The maximum credibility tree was obtained with TreeAnnotator 1.10.4 [49] and visualized using FigTree 1.4 [54].

#### Historical demography analysis

To estimate the demographic dynamics of *L*. *nivalis* over time, we generated Bayesian Skyline plots using BEAST 1.10.4 [49,55,56]. Coalescence times for each paternal lineage were calculated individually, considering all individuals, using GTR+G+I in a Piecewise-linear nucleotide substitution model obtained in jModeltest, as we explained above [49].

Genealogies and model parameters for each lineage were sampled every 50,000 iterations for 5 × 10^8^ generations under a relaxed lognormal molecular clock with uniformly distributed priors and a pre-burn in of 1000 [49]. Demographic plots for each analysis were visualized with Tracer 1.7.1 [53]. To scale the time in the Bayesian coalescence Skyline plot (representing evolutionary time as actual real time in years), we used the last divergence time of the branches as determined in the calibrated tree [49].

## Results

### Genetic diversity

We analyzed a total of 181 *L*. *nivalis* bats: 89 males and 92 females from nine localities across the distribution range of the species (Table 1; Fig 1). We studied three genetic regions: two maternally inherited mitochondrial regions (1,128 bp for *Cyt-b* (n = 154; 95 haplotypes); 828 bp for *D-loop* (n = 144; 28 haplotypes)), and 482 bp from the nuclear Y chromosome *DBY* region in males (n = 69; 44 haplotypes). Total genetic diversity (*Hd*, Table 2) estimates varied across genetic regions. The *D-loop* region showed lower diversity *(Hd* = 0.775) compared to the *Cyt-b* region (*Hd* = 0.937). In contrast, genetic diversity estimates for *Cyt-b* (*Hd* = 0.937) and *DBY* (*DBY*, *Hd* = 0.946) were similar. Haplotype diversity for the mitochondrial markers did not differ significantly among the sampled localities (Table 1), suggesting no apparent bias when comparing localities for each marker separately. However, *DBY* exhibited the highest genetic diversity in three populations from distinct regions across the species’ distribution range (i.e., Texas, USA, in the North, Michoacan in the West, and Morelos, Mexico, in the South), representing the geographic extremes of the distribution of *L*. *nivalis*. In populations with smaller sample sizes, haplotype diversity estimates are equal to 1 (Estado de Mexico, Puebla, and Tonatico, Morelos).

**Table 2:** Genetic diversity for *Leptonycteris nivalis*, including sample sizes and diversity indices for mtDNA, cytochrome B (*Cyt-b*), control region (*D-loop*), and for the Dead box Y-linked (DBY) genes.

	Sample size (n)	Segregating sites (S)	Number of haplotypes	Haplotype diversity (*Hd*)	Nucleotide diversity (π)
*Cyt-b*	153	234	95	0.9376	0.00715
*D-loop*	132	19	28	0.7976	0.00835
*DBY*	69	172	44	0.9463	0.02520

### Population genetic structure

AMOVA analyses for all three markers indicated that genetic variation was lower among populations than within populations (Table 3). For *Cyt-b*, 68.93% of genetic variation is explained by variation within populations, while it was higher for *D-loop* and *DBY*, with values of 91.13% and 82.63%, respectively. Consequently, the *F*_ST_ value for *D-loop* (0.089, *p* = 0.00030) was the lowest among all markers, while *Cyt-b* showed the highest value (0.311, *p* = 0.00000), and *DBY* had an intermediate value (0.174, *p* = 0.00000).

**Table 3:** Analysis of molecular variance (AMOVA) for *Leptonycteris nivalis* in all sampled locations included in Table 2.

*Cyt-b*						
Analysis	Source of variation	df	Sum of squares	Variance components	percentage of variation	*F*-statistic
All populations	Among populations	8	229.168	1.63952	31.07	
	Within populations	144	523.859	3.63791	68.93	*F*_ST_ = 0.31067
	Total	152	753.026	5.27743		p < 0.00030
***D-loop***						
Analysis	Source of variation	df	Sum of squares	Variance components	percentage of variation	*F*-statistic
All populations	Among populations	6	33.833	0.20967	8.87	
	Within populations	125	269.357	2.15485	91.13	*F*_ST_ = 0.08867
	Total	131	303.189	2.36452		p < 0.00001
***DBY***						
Analysis	Source of variation	df	Sum of squares	Variance components	percentage of variation	F-statistic
All populations	Among populations	8	103.815	1.08013	17.37	
	Within populations	60	308.286	5.13811	82.63	*F*_ST_ = 0.17370
	Total	68	412.101	6.21824		p < 0.00001

**Fig 2:**
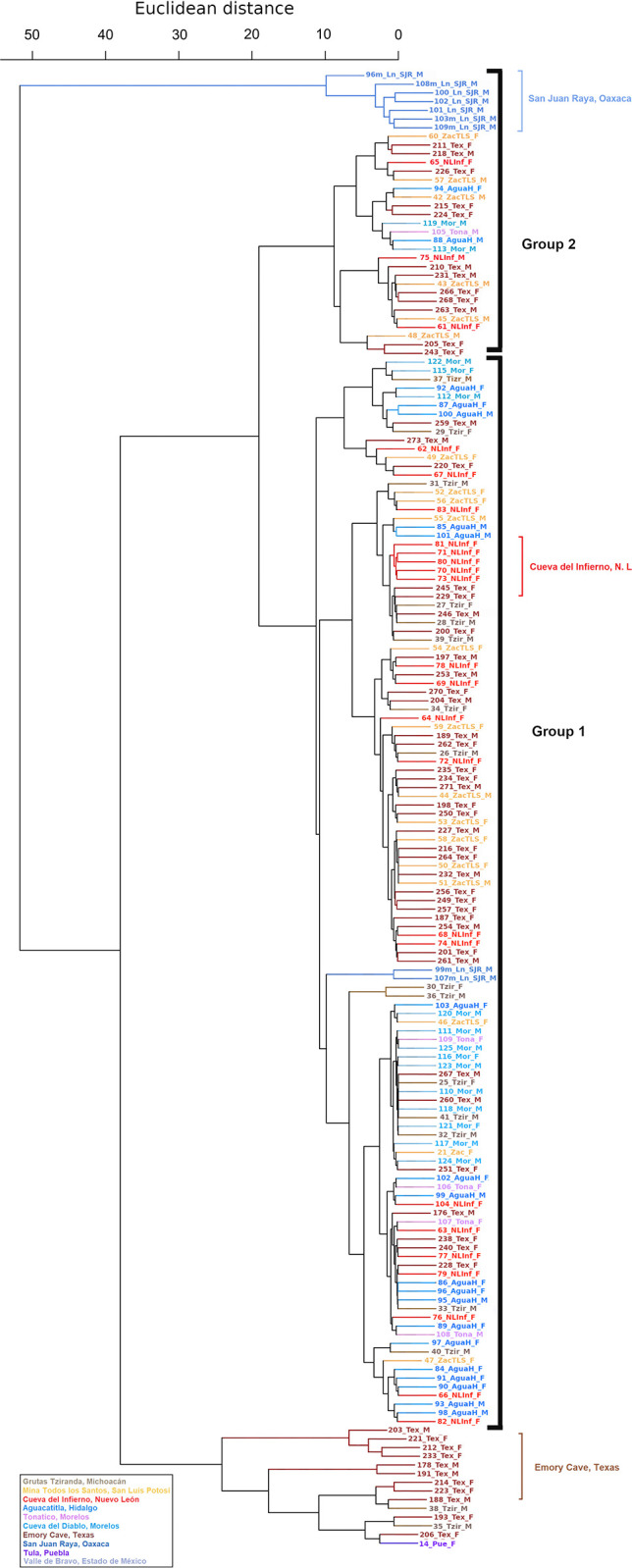
Mitochondrial gene dendrogram based on *Cyt-b* and *D-loop* for *Leptonycteris nivalis*, constructed with Euclidean distances. Black font shows both groups identified by geographic location.

**Fig 3:**
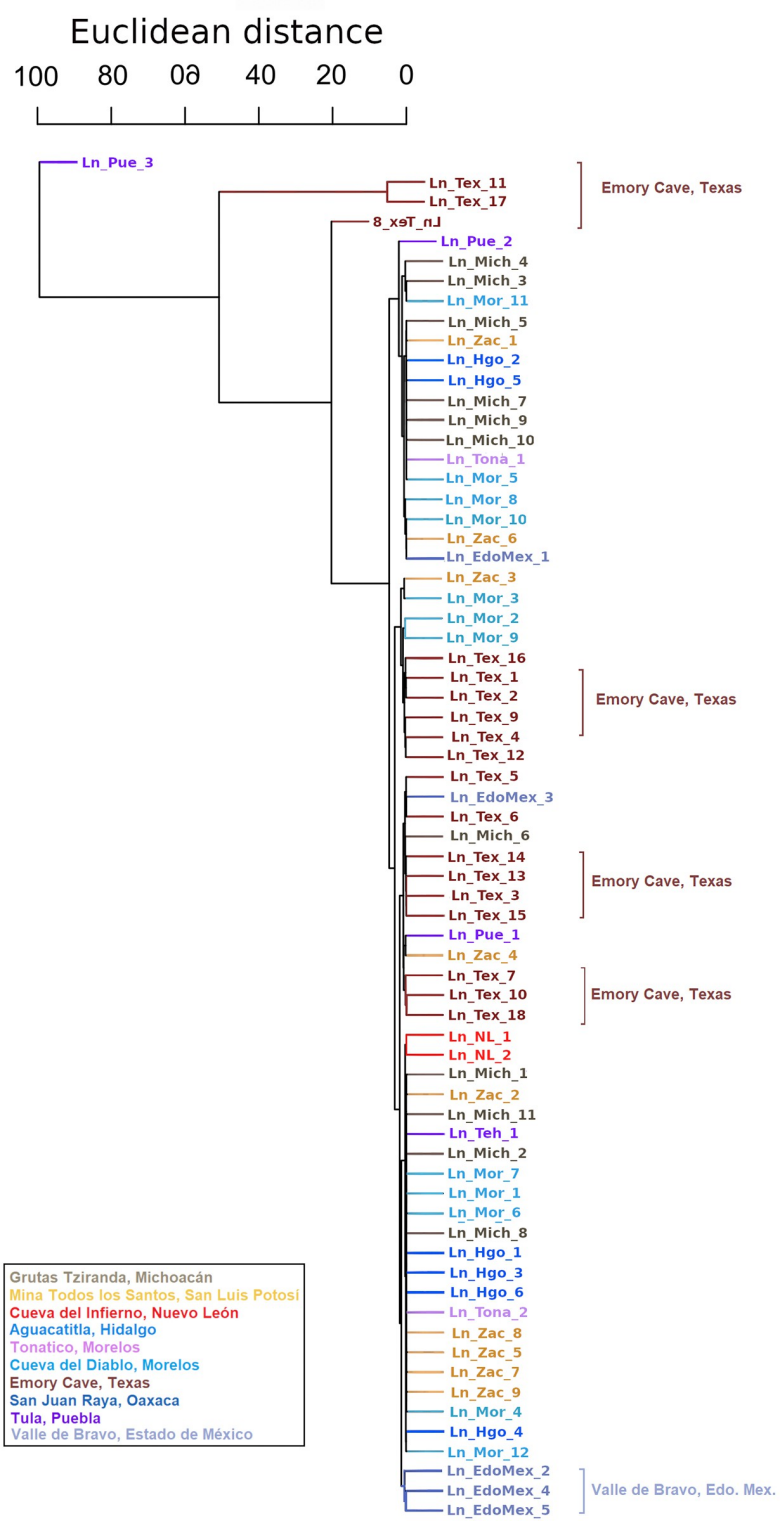
Chromosome-Y associated gene dendrogram based on *DBY* for *Leptonycteris nivalis*, constructed with Euclidean distances.

According to a dendrogram constructed using the mitochondrial Euclidean distances (Fig 2), there is no complete nor clear geographical congruence of groups or sampled localities. However, we observed a distinct group composed of *L*. *nivalis* from Puebla-Oaxaca (from the town of San Juan Raya, SJR thereafter), and another group containing most of the *L*. *nivalis* from Texas (Fig 2). In contrast, in the dendrogram based on the Y chromosome-associated marker (Fig 3), there are no well defined groups.

### Connectivity

**Fig 4:**
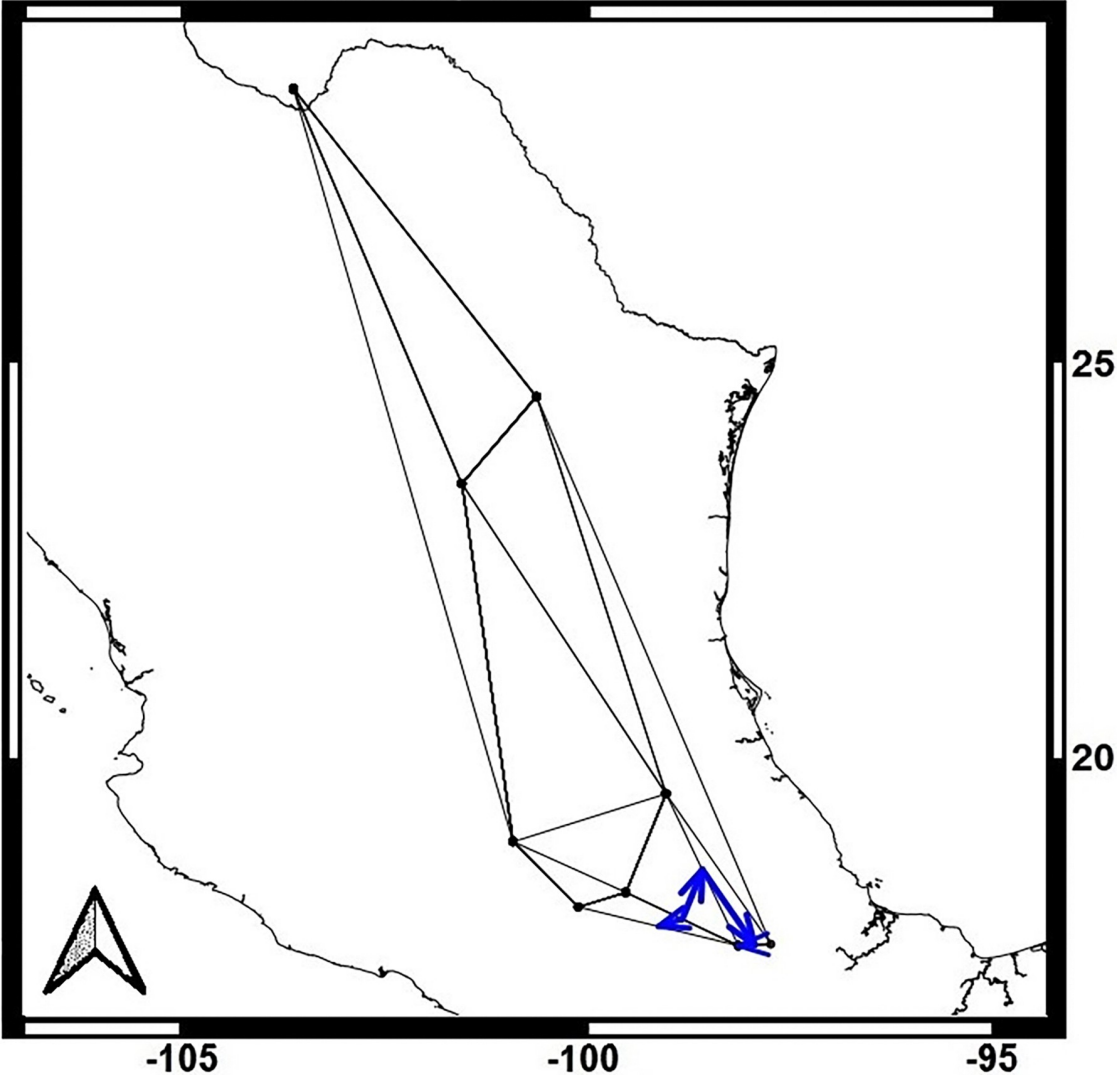
Monmonier’s analyses for the Maternal gene markers *Cyt-b* and *D-loop*. Blue arrows show genetic barriers. Basemap was taken from (CONABIO (http://geoportal.conabio.gob.mx/metadatos/doc/html/dest22gw.html).

**Fig 5:**
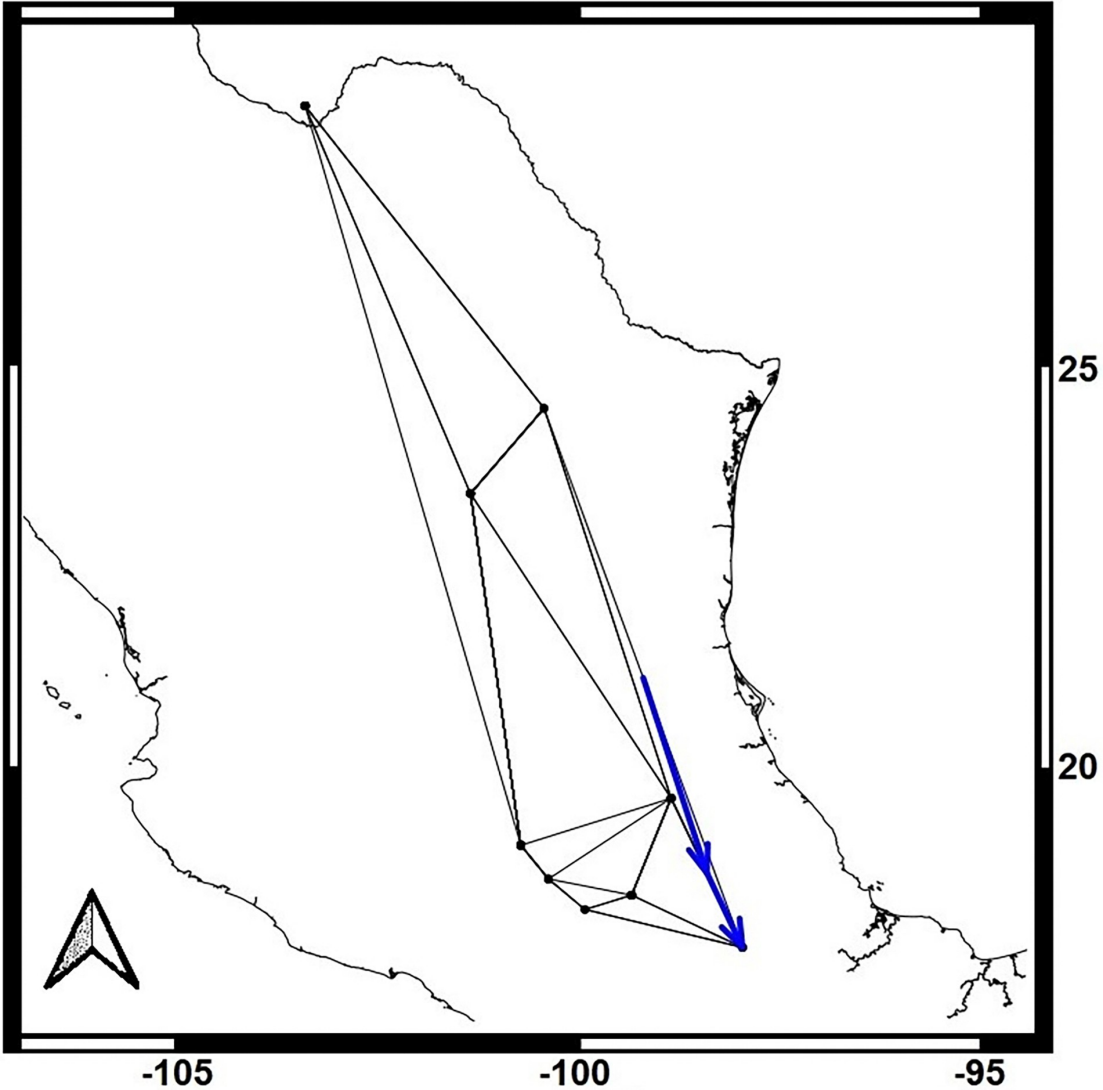
Monmonier’s analyses for the Paternal chromosome-Y associated gene markers *DBY*. Blue arrows show genetic barriers. Basemap was taken from (CONABIO (http://geoportal.conabio.gob.mx/metadatos/doc/html/dest22gw.html).

Monmonier’s function identified genetic barriers for each lineage, indicating isolation in the southern part of the distribution for both lineages, with some differences between maternal and paternal markers (Figs 4 and 5). The optimize.monmonier creates a monmonier object by testing multiple starting points, and returning the optimal boundary [47]. Monmonier’s algorithm aims to find the path with the highest genetic distances between neighboring populations [47]. This analysis is consistent with the AMOVA results, which also detected genetic population structure. In the maternal lineages (Fig 4), a barrier was detected in the southern locations, particularly for Morelos and Puebla-Oaxaca sites, suggesting restricted northward movement for bats from the southern extreme of the distribution. In contrast, the paternal marker (Fig 5), revealed a barrier in the southeast, along the Sierra Madre Oriental, as well as another barrier between the Morelos-Puebla-Oaxaca region and northern and western sites.

### Historical analyses

#### Divergence times

**Fig 6:**
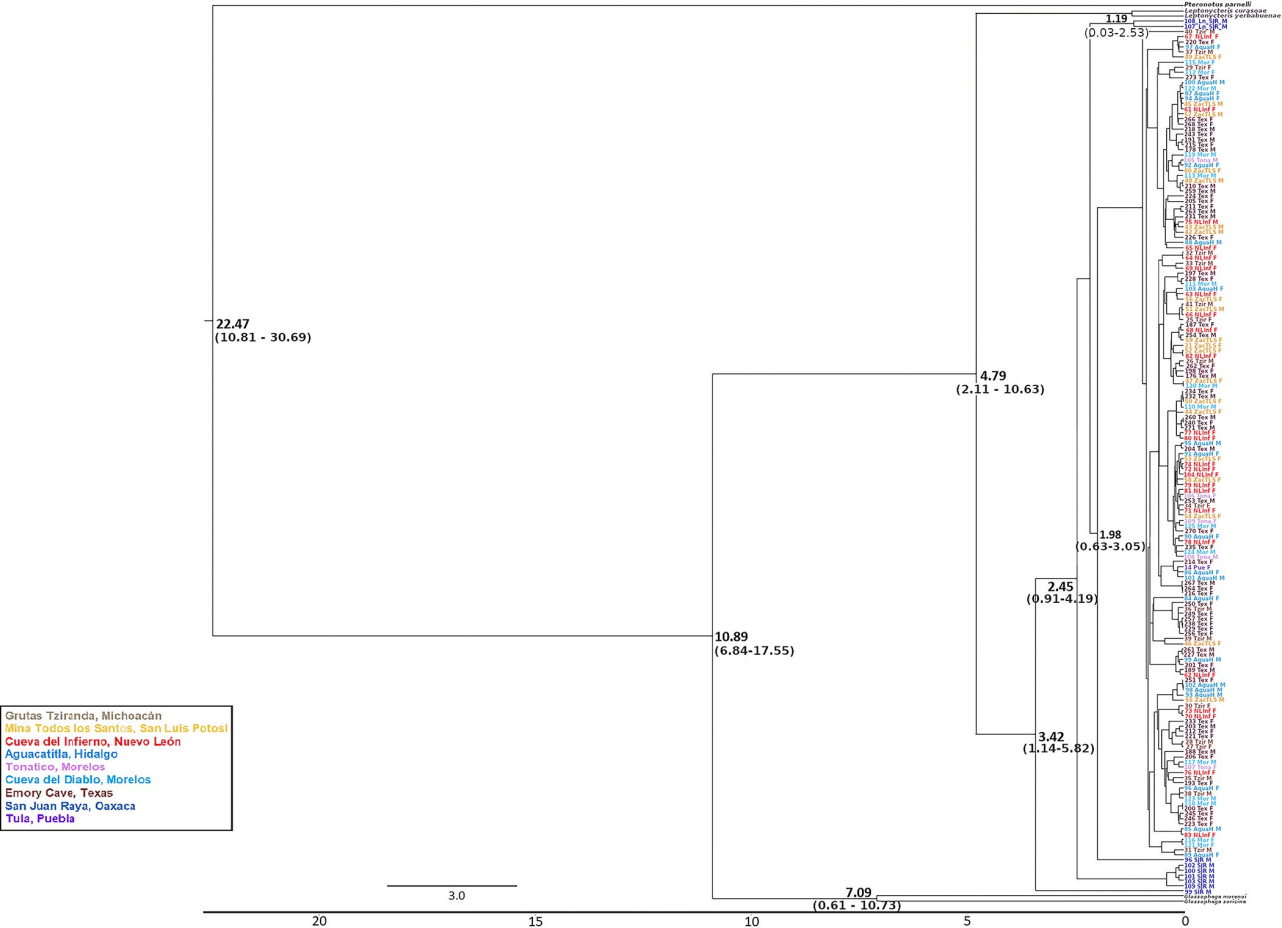
Bayesian mitochondrial gene genealogy based on *Cyt-b and D-loop* for *Leptonycteris nivalis*. Node numbers represent divergence times in thousands of years.

**Fig 7:**
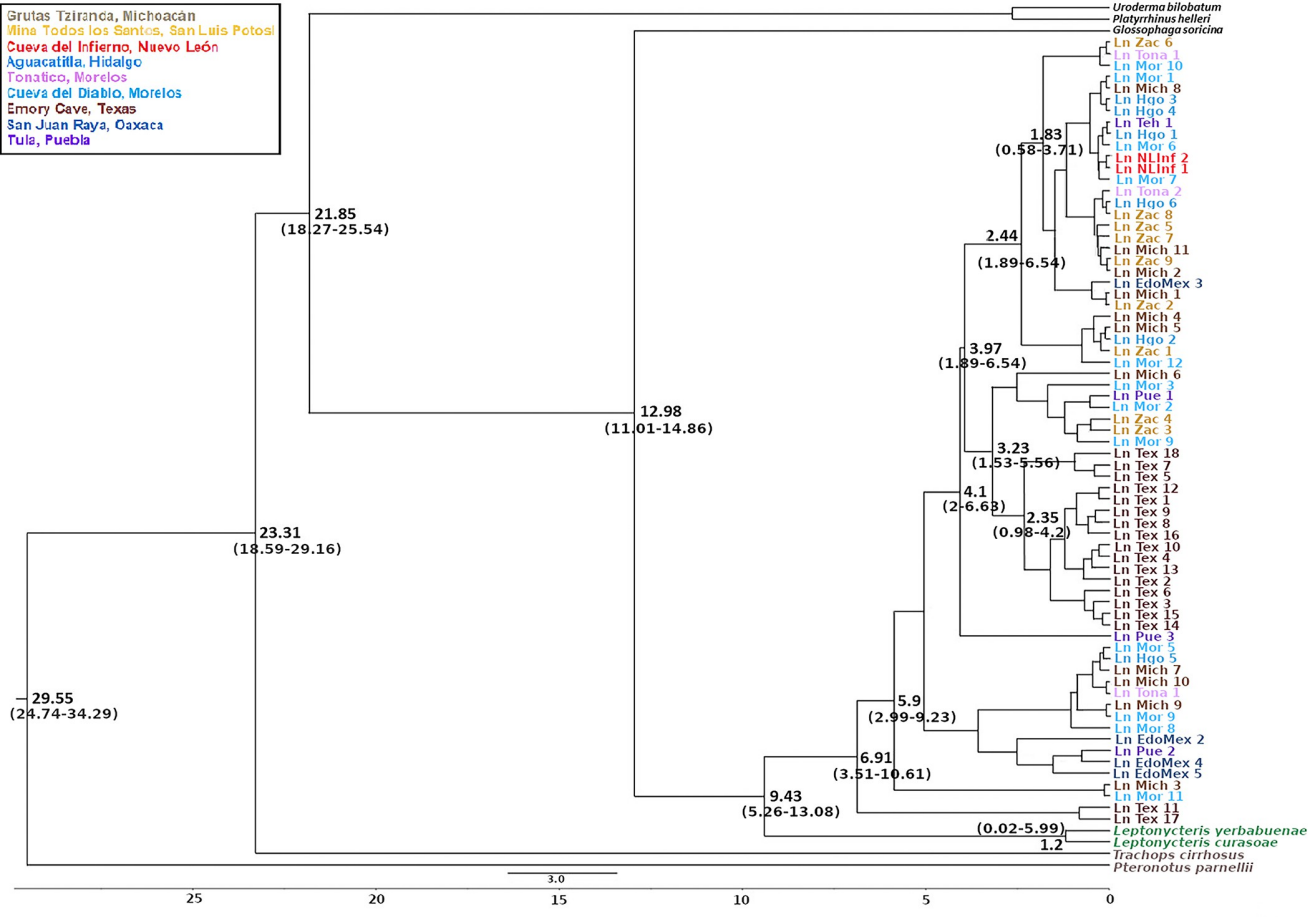
Bayesian gene genealogy based on *DBY* for *Leptonycteris nivalis*; node numbers represent divergence times in thousands of years.

The mitochondrial (*Cyt-b* + *D-loop*) Bayesian chronogram (Fig 6) supports *L*. *nivalis* as monophyletic, including different lineages and estimated that *L*. *nivalis* originated at 3.42 mya (crown group, 95% HDP 1.14–5.82 mya), with a divergence from the other two species in the genus (i.e., *L*. *curasoae* and *L*. *yerbabuenae*) at 4.79 mya (95% HDP 2.11–10.63 mya). For *DBY* (Fig 7), we obtained older dates (6.91 mya for the divergence of the crown group, 95% HDP, 3.51–10.61 mya; 9.43 mya for the divergence from the rest of the species of the genus 95% HD 5.26–13.08 mya). These maternal Bayesian analyses do not show clear geographic congruence; however, two sister groups were identified in the southern part of the species’ distribution. Despite this lack of geographic structure, all groups exhibited short branches, suggesting that the species has undergone a demographic expansion. For the paternal lineages, the *DBY* gene genealogy reveals two monophyletic groups of males from the Texas site.

#### Historical demography analysis

**Fig 8:**
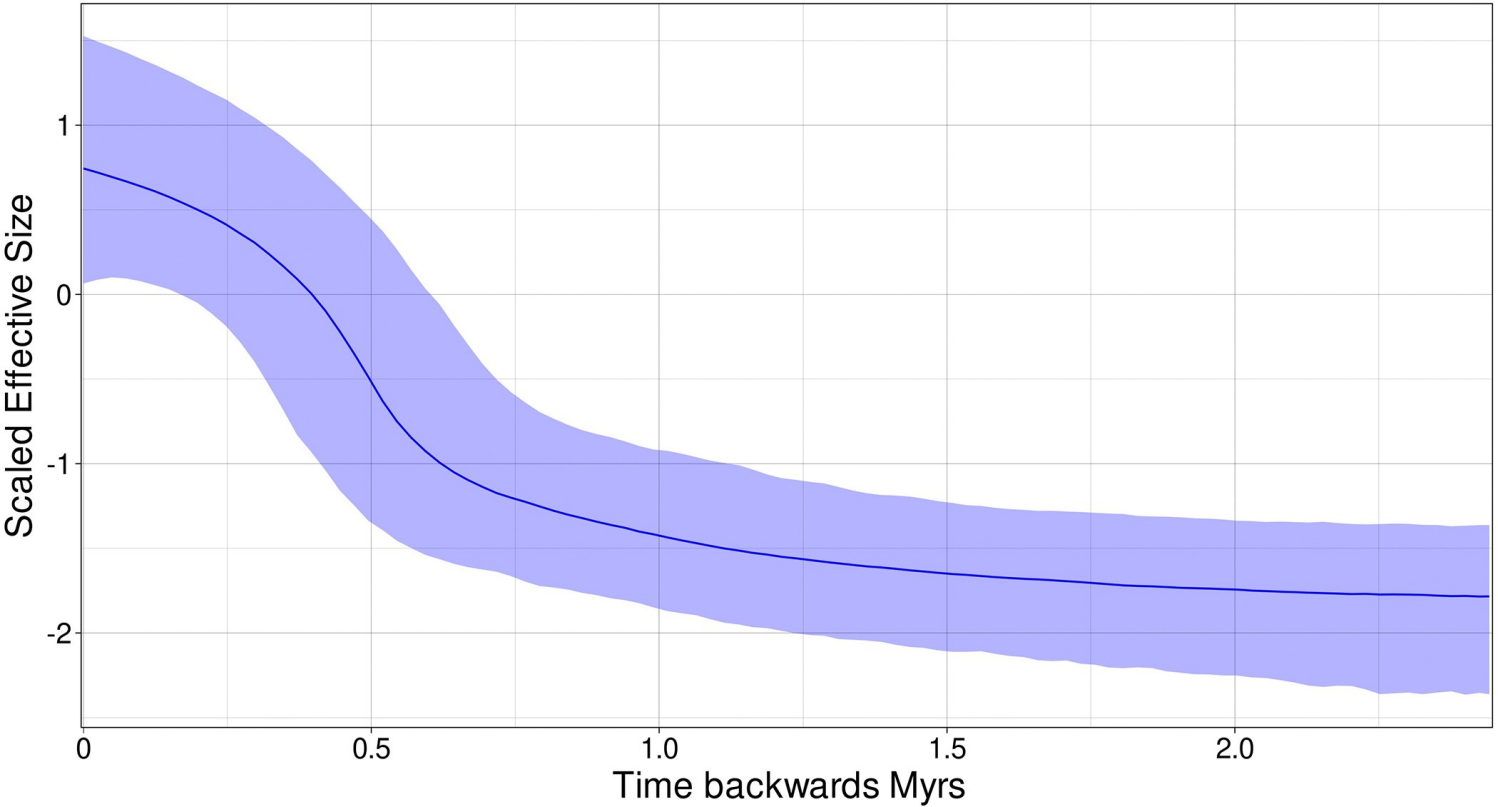
Demographic history Skyline plots generated using BEAST software [49]. These plots simulate demographic changes based on genetic diversity under bayesian algorithms for *Leptonycteris nivalis* estimated for Maternal *Cyt-b- D-loop* sequences. The time is represented in thousands of years (axis X) and changes in scale effective population size (axis Y).

**Fig 9:**
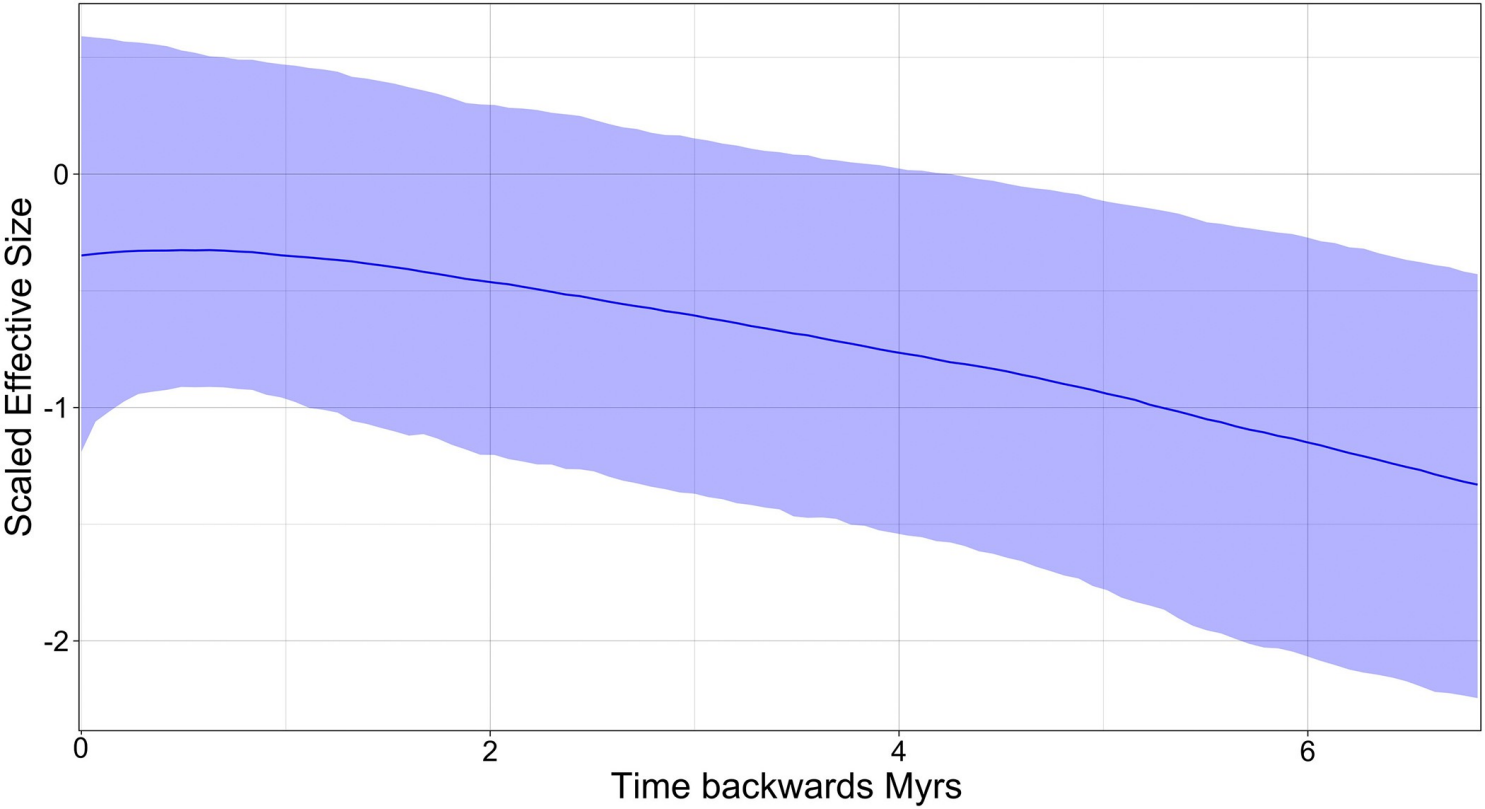
Demographic history Skyline plots generated using BEAST software [49]. These plots simulate demographic changes based on genetic diversity under bayesian algorithms for *Leptonycteris nivalis* estimated for Paternal *DBY* marker. The time is represented in thousands of years (axis X) and changes in scale effective population size (axis Y).

Bayesian Skyline plots for the mitochondrial DNA (Fig 8) indicate a late Pleistocene demographic expansion, starting at ~600,000 years ago. The Bayesian Skyline plot for the paternal *DBY* genes (Fig 9) suggested more stability with an older and constant demographic expansion that began ca. 6–7 mya, coincident with the evolutionary history and divergence times observed in the gene genealogy (Fig 7).

## Discussion

Both the mitochondrial *Cyt-b* and nuclear *DBY* genes exhibited high diversity (*Cyt-b* = 0.93, *DBY* = 0.94), while the *D-loop* region had lower diversity *(Hd* = 0.775). The discrepancies between the two analyzed mitochondrial genes may be attributed to differences in their mutation or substitution rates, and variations in the efficiency of purifying selection [57–59]. Although no clear genetic structure was detected among localities in the paternal lineage analysis, *Fst* values for all three genes ranged from 0.08867 to 0.31067, indicating some genetic differentiation. Additionally, Monmonier’s analyses identified genetic barriers in the southern part of the distribution (i.e., in central Mexico), although these barriers varied slightly in location.

Divergence times calculated for the gene genealogies were consistent with those reported for the sister species, *L*. *yerbabuenae*, across the three genetic markers. Specifically, divergence times were estimated at 4.03 million years ago (mya) for Cyt-b (95% HDP, 2.23–8.15 mya), 4.86 mya for D-loop (95% HDP, 2.156–8.14 mya), and 12.01 mya for DBY (95% HDP, 9.62–13.43 mya) [36], allowing for comparisons with our results. It is important to note that both species exhibit similar migratory behavior and have overlapping distributions in many localities during non-migratory seasons.

In *L*. *nivalis* we found discrepancies between maternal and paternal markers, with more recent divergence dates for the maternal lineage. The divergence age of *L*. *nivalis* from its sister species was estimated at 4.79 mya for the maternal lineage, while for the paternal lineage it was estimated at 9.43 mya, which is similar to a previous estimate for *L*. *yerbabuenae*, using the same genetic marker (*DBY*) [36]. This discrepancy may be explained by differences in sampling size among sites, which could introduce biases in some cases. As we mentioned before, the population sizes of *L*. *yerbabuenae* are considerably larger than those of *L*. *nivalis* and its distribution range is also broader. While *L*. *nivalis* is restricted to central Mexico and the southern United States, female *L*. *nivalis* migrate to Texas during the migratory season. Additionally, there are not many known refuges for this species [30,36].

### Genetic diversity

Our sampling effort included different localities across the entire species distribution, enabling us to explore aspects of the migratory behavior of *L*. *nivalis* through genetic analysis. We believe that our results provide a foundation for understanding how ecological factors have influenced *L*. *nivalis* and that our sampling is adequate and representative for estimating genetic diversity within and among populations using both maternal and paternal genetic markers. Additionally, we were able to increase sampling effort compared to previous studies of the species [13,29,60].

The haplotype diversity for *L*. *nivalis* is in general high, similar to the estimates reported for the closely related species, *L*. *yerbabuenae* [36]. For *Cyt-b*, *L*. *nivalis* exhibited a higher diversity value (*Hd* = 0.9376) than for *L*. *yerbabueanae* (*Hd* = 0.757). In migratory bat species, estimates for mitochondrial haplotype diversity seem to be high in general; for instance, in *Lasiurus borealis* (*Hd* = 0.95, [61], *Tadarida brasiliense* (*Hd* = 0.998; [18] or *Sturnira parvidens* (*Hd* = 0.9; [62]). Regarding the mitochondrial *D-loop* marker and Y-chromosome, *DBY* estimates are similar between *L*. *nivalis* (*Hd D-loop* = 0.7976, *Hd DBY* = 0.9463) and *L*. *yerbabuenae* (*Hd D-loop* = 0.8082, *Hd DBY* = 0.9137; [36]). The high levels of haplotype diversity may be attributed to migratory movements, that are not always philopatric. In some cases, migratory individuals may not return to their original refuges, resulting in continuous genetic flow among localities.

A previous study on *L*. *nivalis* [16] reported a sex bias during spring-summer seasons, with females being more abundant at northern latitudes. Given this observed sex bias we expected differences in genetic diversity due to differential migratory behavior between the sexes. To explore this, we analyzed maternal and paternal lineages separately. However, we found no significant differences in haplotype diversity of the mitochondrial markers across sites. In contrast, the *DBY* marker exhibited the highest genetic diversity in populations at the extreme sites of its distribution: Texas, USA, Puebla, and Morelos in Mexico (Edo-Mex, Pue = 1; Texas = 0.99; Table 1).

The similarity in haplotype diversity estimates among different localities for both maternal and paternal genetic markers in *L*. *nivalis* contrasts with its close relative *L*. *yerbabuenae*, where genetic diversity values are distinctly structured by geographic groups, one representing the Pacific coast of Mexico and the other representing the Central-Southern Mexico [36]. Despite this similarity in haplotype diversity, our analysis of *L*. *nivalis* reveals geographic patterns and differences between genetic maternal and paternal lineages in other aspects, including demographic history, connectivity, and divergence times.

### Genetic structure and connectivity

We observed higher values of genetic differentiation in the pairwise comparisons for *Cyt-b* and *DBY* among most localities compared to those in Puebla, but not for the *D-loop*. Our findings partially align with previous reports for *L*. *nivalis* [12,26], though they contrast with studies on *L*. *yerbabuenae* [9,16,63]. It appears that migratory females may contribute to gene flow among geographic sites, reducing genetic differentiation. This phenomenon of sex-biased migration influencing genetic structure has also been observed in other species, such as the blue whale and Asian elephant [64], sandbar shark, *Carcharhinus plumbeus* [65], and green turtle *Chelonia mydas* [66].

Our analyses (Figs 2 and 3 and *F*_*ST*_*´*s) revealed some geographic structure. The mitochondrial (maternal) analyses (Fig 2) show a well-defined group comprising only individuals from the Tehuacán valley Puebla (depicted in blue). Another partially defined cluster includes most of the bats from Texas, one from Puebla, and two from Michoacán. Other individuals from Texas are found along the dendrogram, suggesting that females from various sites migrate to Texas conforming a maternity roost. The *DBY* dendrogram shows some consistency with the mitochondrial dendrogram, with individuals from Texas dispersed across almost all clusters. However, we observed two groups with members exclusively from Texas. In the Tehuacán Valley, in the south of the distribution, we were able to analyze only four males, and there is a possibility that they form an exclusive group, but a larger sample will be needed to confirm our results.

We recognize the importance of future studies comparing the two parental lineages across sampling sites, incorporating a larger number of individuals and additional nuclear, particularly genomic, data to achieve a more comprehensive understanding.

Previous studies have documented the ability of *Leptonycteris* bats to cover large distances in a single night [9,64]. Given this, it is challenging to attribute genetic structure solely to geographical distances. Nevertheless, differences in mating and birthing seasons in the Southern part of the range [29,60] could explain, at least in part, the results of our Monmonier analysis, in which for both male and female genetic lineages, barriers to gene flow were detected in the Southern area.

As emphasized earlier, expanding the sampling efforts will be important, focusing on increasing and standardizing sample sizes per site, including both reproductive males and females, and exploring additional populations. We acknowledge the potential bias introduced by the Texas samples, where numerous haplotypes may be concentrated, as it is one of the most numerous maternity refuges for the species [29], and there could be individuals coming from most of the refuge sites. Sampling this species in different seasons is challenging, especially during female migration, as the locations of refuges for males and non-migratory females remain unknown. Future studies should account for this bias and aim to improve sampling efforts by considering both migratory and non-migratory seasons.

We can compare our findings to those of the closely related species *L*. *yerbabuenae*, whose genetic structure was also studied using both parental markers and supported by a Bayesian analysis [36]. Given the similar migratory dynamics and distinct reproductive seasons of the two species, based on geographical distribution, we expected to find comparable patterns in their genetic structure. *Leptonycteris yerbabuenae* exhibits genetic structuring in the paternal lineage, attributed to male philopatry and female migration movements during spring-summer season [36]. In contrast, *L*. *nivalis* shows no strong genetic structure.

The disparity in genetic structure between both species raises intriguing questions about the factors influencing their population genetics and warrants further investigation. Our results suggests that the low genetic structure of *L*. *nivalis* may be due to the long-distance movements of most females. It is also possible that female migratory movements are not entirely philopatric, with many females potentially changing refuges upon return. This movement pattern could facilitate allele exchange among locations, making it challenging to detect genetic structure and barriers to gene flow.

### Historical analyses

The genealogical relationships obtained for *L*. *nivalis* and related species are clear and congruent with previously published phylogenies [65], where *L*. *nivalis* is the sister group of *L*. *curasoae* + *L*. *yerbabuenae*. However, there are differences when comparing paternal and maternal inherited molecular markers in *L*. *nivalis* as illustrated in their Bayesian genealogies (Figs 5 and 6). We should also note the differences in sample sizes, as for the mitochondrial genealogy the total sample size was 181 bats, while for the paternally inherited *DBY* genealogy, we could only analyze the male bats (69 individuals), and for instance, in the Tehuacán Valley, we could only analyze four males.

In the mitochondrial Bayesian genealogy, we found three distinct groups comprised solely by Texas bats, while in the *DBY* genealogy there is a large monophyletic group of only Texas animals (Fig 7). These observations are interesting, because the Texas locality (i.e., the Emory cave) is a maternity roost. It has been proposed that females from the entire species’ distribution converge at this location to give birth [52] and it remains uncertain where the studied individuals originally came from.

In the initial divergence (i.e., crown node) of *L*. *nivalis* haplotypes in the mitochondrial genealogy, we observed a cluster exclusively composed of bats from Tehuacán Valley (SJR). This finding aligns with similar patterns identified in the mitochondrial genealogy of *L*. *yerbabuenae* based on the same molecular markers in [36]. This observation is interesting because the Tehuacán Valley has distinctive evolutionary and ecological characteristics, including unique climatic conditions and a rich diversity of agaves and columnar cacti, which are the primary nectar sources for *Leptonycteris* spp. [67]. In addition, this region has been proposed as the possible center of origin of the *Leptonycteris* genus [23,36].

Our analysis of the mitochondrial regions suggested a divergence time of 10.89 (6.84–17.55) mya between *Leptonycteris* spp. and *Glossophaga* spp., and the estimated age of the *Leptonycteris* crown group at 4.79 (2.11–10.63) mya. On the other hand, the paternal genetic marker *DBY* showed slightly overlapping, but older divergence times: for the divergence between *Leptonycteris* ssp. and *Glossophaga* spp. it was estimated at 12.98 (11.01–14.86) mya ago, while for the crown group *Leptonycteris*, the date of divergence was 9.43 (5.26–13.08) mya. The paternal genealogy places the crown group of *Leptonycteris* 5 mya older, while the divergence time between *Leptonycteris* and *Glossophaga* differs by 2 mya between the maternal and the paternal genetic markers. These results may be the reflection of different evolutionary histories for each gene, such as differences in mutation and substitution rates and-or efficiency of purifying selection [36,57], as well as variation in the evolutionary history, in particular differences in migration and effective population size, of each parental lineage. A similar discordance was reported for the same molecular markers in *L*. *yerbabuenae* [36], and, for instance, some authors have established that in general the Y-chromosome genes are less polymorphic than mitochondrial genes, as a reflection of their lower mutation rates [66].

The estimated divergence times are consistent with those proposed for *L*. *yerbabuenae* [36], indicating ecological and evolutionary coincidences between *Leptonycteris* bats, and in particular between *L*. *nivalis*, and the plant genus *Agave*. Notably, the origin of *Agave sensu lato* (4.6–12.3 mya [52]; and a reported 6.18 mya crown age for *Agave* [68]) mirrors in part the divergence times of *L*. *nivalis* crown group (mitochondrial markers genealogy– 3.42 mya and DBY genealogy– 6.91 mya) [6]. Additionally, the time of divergence for the basal group of *Leptonycteris* for the *DBY* genealogy correlates with two periods of accelerated diversification rate in *Agave sensu lato*, first occurring 8–6 mya, and the second at 3–2.5 mya, according to [69], and reported at 6.18 mya [69] and the second at 4.91 mya [68]. Moreover, these divergence times coincide with a temperature decrease for tropical wet climates in Mexico during glacial periods, between 5.3 and 1.8 mya [70].

We identified a correlation between the demographic history of populations and the structure of ultrametric trees in both maternal and paternal genealogies. Our genealogies and Skyline plot analyses likely reflect a historic demographic expansion, apparently linked to Pleistocene climate changes, but when compared with *L*. *yerbabuenae*, our analysis showed an older demographic expansion in *L*. *nivalis*. During the Pleistocene, both *L*. *nivalis* (this study) and *L*. *yerbabuenae* [36] underwent geographic and demographic expansions, apparently erasing in both species the signals of previous geographic structure. Demographic analyses also revealed a significant difference between parental lineages, indicating a demographic expansion for the female inherited markers (which migrate to the north), but not in the male lineage (which do not migrate).

This demographic expansion is supported by past projection models for *L*. *nivalis* [23], where climatic scenarios reveal a historical (Pleistocene) and current geographic congruence between Mexican *Leptonycteris* bats and several *Agave* species. Additionally, due to high haplotype diversity and medium-low nucleotide diversity, along with the Skyline plots for mtDNA, a demographic expansion in *L*. *nivalis* around 600,000–700,000 years ago, during the Pleistocene is suggested. These results are further supported by past distribution models showing a geographic expansion from the LIG through the Holocene reported for the species and an overlapping with some of its most important sources of food, plants of the genus *Agave* [23]. We predicted that migratory movements observed in the present are a result of a recent geographic expansion prompted by changes that produced new suitable climatic and ecological conditions for the species.

The effects of Pleistocene climate changes on species distribution have been well documented in different studies. In particular, the decrease in temperature during interglacial periods led to the fragmentation of the Mexican highland biota [67,71–74], while subsequent increases in temperature resulted in geographic and demographic expansions [75–77]. We believe that the present distribution and historical demography of *L*. *nivalis* were likely shaped by these climate shifts. Remarkably, the current distribution of *L*. *nivalis* aligns with several species of the genus *Agave*, suggesting similar historical dynamics (see [25,30,78]). Furthermore, currently, there exists a strong mutualistic ecological relationship between *L*. *nivalis* and different *Agave* species [11].

Based on our analysis and past-present distribution models, we believe that the migration of females of *L*. *nivalis* to a northern maternal roost has been an ecological and dynamic process (more or less recent) originating from their southern distribution area (Puebla-Oaxaca, SRJ; Fig 1). Our analysis of genetic diversity and Bayesian genealogies, as well as our interpretation of the analyses of the three markers, suggest that the species’ origin could have occurred in the south of its present-day distribution, around the SJR area, as it was proposed before [20], and where *L*. *nivalis* and *L*. *yerbabuenae* are still sympatric [23,36]. The basal lineage in the mitochondrial gene genealogy may correspond with an ancestor of the current populations of *L*. *nivalis* that once existed in the southern part of its current distribution. Nonetheless, for a more comprehensive understanding, it will be important, as stated above, to increase sample sizes and incorporate more robust genomic data, coalescence analyses, and simulations, including all three extant species of *Leptonycteris*.

### Conclusions and perspectives for the conservation of the species

*Leptonycteris nivalis* exhibits high levels of genetic variation, both in mitochondrial and Y-chromosome *DBY* gene analyses. The observed low genetic structure suggests that migrant females play a crucial role in facilitating gene flow, contributing to the cohesiveness of the population. Our finding of genetic barriers in the southern distribution may be associated with male philopatry, which could lead to genetic differentiation in the future. Our demographic analyses revealed contrasting patterns between parental lineages: while there is evidence of demographic expansion for female-inherited molecular markers, this was not observed for male inherited *DBY*. Despite these differences, it is relevant to consider the potential influence of geological and ecological processes on expansion over the last ~ 600,000 years. We anticipated that our analyses would reveal strong genetic connectivity among localities in the species, which is indeed what we documented in this study.

For the conservation of *L*. *nivalis* it will be essential to maintain genetic connectivity and to promote strategies that support its ecological role as a primary pollinator of both wild and cultivated *Agave* plants, as well as wild columnar cacti (typically not planted and seldom managed) across the Chihuahuan desert and surrounding regions in North and Central Mexico [5,79–81]. All of this will become critical in climate change scenarios, yet our understanding of the effects of climate change on bats is still incipient [see 82]. To ensure the future of this and other species, carefully planned, and detailed molecular genetics and genomics studies are essential. Additionally, our work has the potential to support current conservation programs focused on preserving agaves in key ecological and economic areas, while facilitating the unrestricted migratory movements of bats [2,83].

Currently, *Leptonycteris nivalis* faces a much greater extinction risk than *L*. *yerbabuenae*, which was delisted in Mexico in 2013 and in the U.S. in 2017 ([9,84,85]). The eventual recovery of *L*. *nivalis* depends not only on site-specific conservation efforts but also, given its cross-border, migratory movements, the reciprocal, telecoupled ecosystem services provided on one side of the Mexico-U.S. border depend on the conservation actions on the other side [86]. These spatial subsidies are the best way to ensure cooperation for the conservation of this and many other migratory species.

## References

[1] Kunz TH, editor. Ecology of Bats. Boston, MA: Springer US; 2013. doi: 10.1007/978-1-4613-3421-7

[2] Trejo-Salazar R-E, Eguiarte LE, Suro-Piñera D, Medellin RA. Save Our Bats, Save Our Tequila: Industry and Science Join Forces to Help Bats and Agaves. Natural Areas Journal. 2016;36: 523–530. doi: 10.3375/043.036.0417

[3] Borbón-Palomares DB, Laborin-Sivirian F, Tinoco-Ojanguren C, Peñalba MC, Reyes-Ortega I, Molina-Freaner F. Reproductive ecology of *Agave colorata*: the importance of nectar-feeding bats and the germination consequences of self-pollination. Plant Ecology. 2018;219: 927–939. doi: 10.1007/s11258-018-0847-x

[4] Frick WF, Heady PA, Earl AD, Arteaga MC, Cortés-Calva P, Medellín RA. Seasonal ecology of a migratory nectar-feeding bat at the edge of its range. Journal of Mammalogy. 2018;99: 1072–1081. doi: 10.1093/jmammal/gyy088 30323407 PMC6178787

[5] Sánchez R, Medellín RA. Food habits of the threatened bat *Leptonycteris nivalis* (Chiroptera: Phyllostomidae) in a mating roost in Mexico. Journal of Natural History. 2007;41: 1753–1764. doi: 10.1080/00222930701483398

[6] Eguiarte LE, Jiménez Barrón OA, Aguirre‐Planter E, Scheinvar E, Gámez N, Gasca‐Pineda J, et al. Evolutionary ecology of *Agave*: distribution patterns, phylogeny, and coevolution (an homage to Howard S. Gentry). American Journal of Botany, (2021) 108(2), 216–235. doi: 10.1002/ajb2.1609 33576061

[7] Ayala-Berdon J, Galicia R, Flores-Ortíz C, Medellín RA, Schondube JE. Digestive capacities allow the Mexican long-nosed bat (*Leptonycteris nivalis*) to live in cold environments. Comparative Biochemistry and Physiology Part A: Molecular & Integrative Physiology. 2013;164: 622–628. doi: 10.1016/j.cbpa.2013.01.015 23370293

[8] Hensley AP, Wilkins KT. *Leptonycteris nivalis*. Mammalian Species. 1988; 1. doi: 10.2307/3504229

[9] Medellin RA, Rivero M, Ibarra A, De La Torre JA, Gonzalez-Terrazas TP, Torres-Knoop L, et al. Follow me: foraging distances of *Leptonycteris yerbabuenae* (Chiroptera: Phyllostomidae) in Sonora determined by fluorescent powder. Journal of Mammalogy. 2018;99: 306–311. doi: 10.1093/jmammal/gyy016

[10] IUCN. The IUCN Red List of Threatened Species. Version 2016–1. Available at: www.iucnredlist.org. (Accessed: 10 November 2022). 2016.

[11] Moreno-Valdez Arnulfo, Honeycutt Rodney L., Grant William E. Colony Dynamics of *Leptonycteris nivalis* (Mexican Long-Nosed Bat) Related to Flowering *Agave* in Northern Mexico. Journal of Mammalogy. 2004 85(3), 453–459. doi: 10.1644/1383942

[12] Gómez-Ruiz EP, Lacher TE. Modelling the potential geographic distribution of an endangered pollination corridor in Mexico and the United States. Van Kleunen M, editor. Diversity and Distribution. 2017;23: 67–78. doi: 10.1111/ddi.12499

[13] Pourshoushtari RD, Ammerman LK. Genetic variability and connectivity of the Mexican long-nosed bat between two distant roosts. Journal of Mammalogy. 2021;102: 204–219. doi: 10.1093/jmammal/gyaa138

[14] Moreno-Valdez A, Grant WE, Honeycutt RL. A simulation model of Mexican long-nosed bat (*Leptonycteris nivalis*) migration. Ecological Modelling. 2000;134: 117–127. doi: 10.1016/S0304-3800(00)00253-2

[15] Burke RA, Frey JK, Ganguli A, Stoner KE. Species distribution modelling supports “nectar corridor” hypothesis for migratory nectarivorous bats and conservation of tropical dry forest. Diversity and Distribution. 2019;25: 1399–1415. doi: 10.1111/ddi.12950

[16] Ammerman L, McDonough M, Hristov N, Kunz T. Census of the endangered Mexican long-nosed bat *Leptonycteris nivalis* in Texas, USA, using thermal imaging. Endangered Species Research. 2009;8: 87–92. doi: 10.3354/esr00169

[17] Moussy C, Hosken DJ, Mathews F, Smith GC, Aegerter JN, Bearhop S. Migration and dispersal patterns of bats and their influence on genetic structure. Mammal Review. 2013;43: 183–195. doi: 10.1111/j.1365-2907.2012.00218.x

[18] Russell AL, Medellín RA, Mccracken GF. Genetic variation and migration in the Mexican free-tailed bat (Tadarida brasiliensis mexicana): Population Genetics of *Tadarida brasiliensis*. Molecular Ecology. 2005;14: 2207–2222. doi: 10.1111/j.1365-294X.2005.02552.x 15910338

[19] Graham Brendan A., Burg Theresa M. Molecular markers provide insights into contemporary and historic gene flow for a non-migratory species. Journal of Avian Biology. 2 May 2012; 43: 198–214.

[20] Ripperger SP, Tschapka M, Kalko EKV, Rodriguez-Herrera B, Mayer F. Life in a mosaic landscape: anthropogenic habitat fragmentation affects genetic population structure in a frugivorous bat species. Conservation Genetics. 2013;14: 925–934. doi: 10.1007/s10592-012-0434-y

[21] Meyer CFJ, Kalko EKV, Kerth G. Small-Scale Fragmentation Effects on Local Genetic Diversity in Two Phyllostomid Bats with Different Dispersal Abilities in Panama. Biotropica. 2009;41: 95–102. doi: 10.1111/j.1744-7429.2008.00443.x

[22] Aguirre-Planter E, Parra-Leyva JG, Ramírez-Barahona S, Scheinvar E, Lira-Saade R, Eguiarte LE. Phylogeography and Genetic Diversity in a Southern North American Desert: Agave kerchovei From the Tehuacán-Cuicatlán Valley, Mexico. Frontiers in Plant Sciences. 2020;11: 863. doi: 10.3389/fpls.2020.00863 32733498 PMC7358651

[23] Trejo-Salazar Roberto-Emiliano. Filogeografía y Conservación del Murciélago Magueyero Menor, *Leptonycteris yerbabuenae* (Martínez Y Villa 1940). CDMX, México: Universidad Nacional Autónoma de México; 2023.

[24] Ramírez‐Barahona S, Eguiarte LE. The role of glacial cycles in promoting genetic diversity in the Neotropics: the case of cloud forests during the Last Glacial Maximum. Ecology and Evolution. 2013;3: 725–738. doi: 10.1002/ece3.483 23531632 PMC3605859

[25] Scheinvar E, Gámez N, Castellanos-Morales G, Aguirre-Planter E, Eguiarte LE. Neogene and Pleistocene history of *Agave lechuguilla* in the Chihuahuan Desert. Journal of Biogeography. 2017;44: 322–334. doi: 10.1111/jbi.12851

[26] Hipp AL, Manos PS, González‐Rodríguez A, Hahn M, Kaproth M, McVay JD, et al. Sympatric parallel diversification of major oak clades in the Americas and the origins of Mexican species diversity. New Phytologist. 2018;217: 439–452. doi: 10.1111/nph.14773 28921530

[27] Gutiérrez-García TA, Vázquez-Domínguez E. Consensus between genes and stones in the biogeographic and evolutionary history of Central America. Quaternary Research. 2013;79: 311–324. doi: 10.1016/j.yqres.2012.12.007

[28] Castellanos-Morales G, Gámez N, Castillo-Gámez RA, Eguiarte LE. Peripatric speciation of an endemic species driven by Pleistocene climate change: The case of the Mexican prairie dog (*Cynomys mexicanus*). Molecular Phylogenetics and Evolution. 2016; 94: 171–181. doi: 10.1016/j.ympev.2015.08.027 26343460

[29] Ammerman Loreen K., Brown Carson, Medellín Rodrigo A., Moreno-Valdez Arnulfo, Pfau R. S., Lesagonicz R, and Russell Amy L. Genetic variation and structure in the endangered Mexican long-nosed bat (*Leptonycteris nivalis*): mitochondrial and nuclear perspectives. From field to laboratory: a memorial volume in honor of Robert J Baker. Special Publications, Museum of Texas Tech University; 2019. pp. 169–185.

[30] Trejo‐Salazar RE, Gámez N, Escalona‐Prado E, Scheinvar E, Medellín RA, Moreno‐Letelier A, et al. Historical, temporal and geographic dynamism of the interaction between *Agave* and *Leptonycteris* nectar‐feeding bats. American J of Botany. 2023; ajb2.16222. doi: 10.1002/ajb2.16222 37561648

[31] Sikes RS, the Animal Care and Use Committee of the American Society of Mammalogists. 2016 Guidelines of the American Society of Mammalogists for the use of wild mammals in research and education: Journal of Mammalogy. 2016; 97: 663–688. doi: 10.1093/jmammal/gyw078 29692469 PMC5909806

[32] Prugnolle F, De Meeus T. Inferring sex-biased dispersal from population genetic tools: a review. Heredity. 2002;88: 161–165. doi: 10.1038/sj.hdy.6800060 11920116

[33] Gasca-Pineda Jaime. Genética de la conservación del bisonte de la pradera (*Bison bison bison*) y del borrego cimarrón (*Ovis canadensis*) en México. CDMX, México; 2015.

[34] Sbisà E, Tanzariello F, Reyes A, Pesole G, Saccone C. Mammalian mitochondrial D-loop region structural analysis: identification of new conserved sequences and their functional and evolutionary implications. Gene. 1997;205: 125–140. doi: 10.1016/s0378-1119(97)00404-6 9461386

[35] Clare EL. Cryptic Species? Patterns of Maternal and Paternal Gene Flow in Eight Neotropical Bats. Stanyon R, editor. PLoS ONE. 2011;6: e21460. doi: 10.1371/journal.pone.0021460 21814545 PMC3144194

[36] Trejo-Salazar R-E, Castellanos-Morales G, Hernández-Rosales D, Gámez N, Gasca-Pineda J, Morales Garza MR, Medellin R and Eguiarte LE. Discordance in maternal and paternal genetic markers in lesser long-nosed bat *Leptonycteris yerbabuenae*, a migratory bat: recent expansion to the North and male phylopatry. PeerJ. 2021;9: e12168. doi: 10.7717/peerj.12168 34703665 PMC8487242

[37] Steppan SJ, Akhverdyan MR, Lyapunova EA, Fraser DG, Vorontsov NN, Hoffmann RS, et al. Molecular Phylogeny of the Marmots (Rodentia: Sciuridae): Tests of Evolutionary and Biogeographic Hypotheses. Systematic Biology. 1999;48: 715–734. doi: 10.1080/106351599259988 12066297

[38] Oshida T, Ikeda K, Yamada K, Masuda R. Phylogeography of the Japanese Giant Flying Squirrel, *Petaurista leucogenys*, Based on Mitochondrial DNA Control Region Sequences. Zoological Science. 2001;18: 107–114. doi: 10.2108/zsj.18.107

[39] Lim BK. Divergence times and origin of neotropical sheath-tailed bats (Tribe Diclidurini) in South America. Molecular Phylogenetics and Evolution. 2007;45: 777–791. doi: 10.1016/j.ympev.2007.09.003 17937995

[40] Lim BK, Engstrom MD, Bickham JW, Patton JC. Molecular phylogeny of New World sheath-tailed bats (Emballonuridae: Diclidurini) based on loci from the four genetic transmission systems in mammals: Phylogeny of new world emballonurid bats. Biological Journal of the Linnean Society. 2007;93: 189–209. doi: 10.1111/j.1095-8312.2007.00942.x

[41] Ewing B, Green P. Base-Calling of Automated Sequencer Traces Using Phred. II. Error Probabilities. Genome Res. 1998;8: 186–194. doi: 10.1101/gr.8.3.186 9521922

[42] Gordon D. Viewing and Editing Assembled Sequences Using Consed. CP in Bioinformatics. 2003;2. doi: 10.1002/0471250953.bi1102s02 18428695

[43] Thompson J. The CLUSTAL_X windows interface: flexible strategies for multiple sequence alignment aided by quality analysis tools. Nucleic Acids Research. 1997;25: 4876–4882. doi: 10.1093/nar/25.24.4876 9396791 PMC147148

[44] Librado P, Rozas J. DnaSP v5: a software for comprehensive analysis of DNA polymorphism data. Bioinformatics. 2009;25: 1451–1452. doi: 10.1093/bioinformatics/btp187 19346325

[45] Excoffier L, Lischer HEL. Arlequin suite ver 3.5: a new series of programs to perform population genetics analyses under Linux and Windows. Molecular Ecology Resources. 2010;10: 564–567. doi: 10.1111/j.1755-0998.2010.02847.x 21565059

[46] Jombart T. *adegenet*: a R package for the multivariate analysis of genetic markers. Bioinformatics. 2008;24: 1403–1405. doi: 10.1093/bioinformatics/btn129 18397895

[47] Manni F, Guerard E, Heyer E. Geographic Patterns of (Genetic, Morphologic, Linguistic) Variation: How Barriers Can Be Detected by Using Monmonier’s Algorithm. Human Biology. 2004;76: 173–190. doi: 10.1353/hub.2004.0034 15359530

[48] Posada D. jModelTest: Phylogenetic Model Averaging. Molecular Biology and Evolution. 2008;25: 1253–1256. doi: 10.1093/molbev/msn083 18397919

[49] Suchard MA, Lemey P, Baele G, Ayres DL, Drummond AJ, Rambaut A. Bayesian phylogenetic and phylodynamic data integration using BEAST 1.10. Virus Evolution. 2018;4. doi: 10.1093/ve/vey016 29942656 PMC6007674

[50] Teeling EC, Springer MS, Madsen O, Bates P, O’Brien SJ, Murphy WJ. A Molecular Phylogeny for Bats Illuminates Biogeography and the Fossil Record. Science. 2005;307: 580–584. doi: 10.1126/science.1105113 15681385

[51] Czaplewski Nicolas J, Takai Masanaru, Naeher Tiffany M., Shigehara Nobuo, Setoguchi Takeshi. Additional bats from the middle Miocene la Venta fauna of Colombia. Revista de la Academia Colombiana de Ciencias Exactas Físicas y Naturales. 2003;27: 263–282.

[52] Flores-Abreu IN, Trejo-Salazar RE, Sánchez-Reyes LL, Good SV, Magallón S, García-Mendoza A, et al. Tempo and mode in coevolution of *Agave sensu lato* (Agavoideae, Asparagaceae) and its bat pollinators, Glossophaginae (Phyllostomidae). Molecular Phylogenetics and Evolution. 2019;133: 176–188. doi: 10.1016/j.ympev.2019.01.004 30639765

[53] Rambaut A, Drummond AJ, Xie D, Baele G, Suchard MA. Posterior Summarization in Bayesian Phylogenetics Using Tracer 1.7. Susko E,. Systematic Biology. 2018;67: 901–904. doi: 10.1093/sysbio/syy032 29718447 PMC6101584

[54] Rambaut A. FigTree v.1.4.4. Computer program distributed by the author. Edinburg: Institute of Evolutionary Biology, University of Edinburgh.: Accessed March 10, 2023.

[55] Ho SYW, Shapiro B. Skyline‐plot methods for estimating demographic history from nucleotide sequences. Molecular Ecology Resources. 2011;11: 423–434. doi: 10.1111/j.1755-0998.2011.02988.x 21481200

[56] Gattepaille LM, Jakobsson M, Blum MG. Inferring population size changes with sequence and SNP data: lessons from human bottlenecks. Heredity. 2013;110: 409–419. doi: 10.1038/hdy.2012.120 23423148 PMC3630807

[57] Allio R, Donega S, Galtier N, Nabholz B. Large Variation in the Ratio of Mitochondrial to Nuclear Mutation Rate across Animals: Implications for Genetic Diversity and the Use of Mitochondrial DNA as a Molecular Marker. Molecular Biology and Evolution. 2017;34: 2762–2772. doi: 10.1093/molbev/msx197 28981721

[58] Nabholz B, Glemin S, Galtier N. Extreme Variation of mtDNA Neutral Substitution Rate across Mammalian Species—the Longevity Hypothesis. Molecular Biology and Evolution. 2008;25: 795–795. doi: 10.1093/molbev/msn033 17998254

[59] Jazin E, Soodyall H, Jalonen P, Lindholm E, Stoneking M, Gyllensten U. Mitochondrial mutation rate revisited: hot spots and polymorphism. Nature Genetics. 1998;18: 109–110. doi: 10.1038/ng0298-109 9462737

[60] Brown, Carson. Natural History and Population Genetics of the Endangered Mexican Long-nosed Bat, *Leptonycteris nivalis* (Chiroptera: Phyllostomidae). M.S. Thesis. San Angelo, Texas, United States of America: Angelo State University; 2004.

[61] Korstian JM, Hale AM, Williams DA. Genetic diversity, historic population size, and population structure in 2 North American tree bats. Journal of Mammalogy. 2015;96: 972–980. doi: 10.1093/jmammal/gyv101

[62] Hernández-Canchola G, León-Paniagua L. Genetic and ecological processes promoting early diversification in the lowland Mesoamerican bat Sturnira parvidens (Chiroptera: Phyllostomidae). Molecular Phylogenetics and Evolution. 2017;114: 334–345. doi: 10.1016/j.ympev.2017.06.015 28647618

[63] Rojas-Martínez A, Valiente-Banuet A, Del Coro Arizmendi M, Alcántara-Eguren A, Arita HT. Seasonal distribution of the long-nosed bat (*Leptonycteris curasoae*) in North America: does a generalized migration pattern really exist? Journal of Biogeography. 1999;26: 1065–1077. doi: 10.1046/j.1365-2699.1999.00354.x

[64] Simal F, De Lannoy C, García-Smith L, Doest O, De Freitas JA, Franken F, et al. Island–island and island–mainland movements of the Curaçaoan long-nosed bat, *Leptonycteris curasoae*. Journal of Mammalogy. 2015;96: 579–590. doi: 10.1093/jmammal/gyv063

[65] Wetterer AL, Rockman MV, Simmons NB. Phylogeny of phyllostomid bats (mammalia: chiroptera): data from diverse morphological systems, sex chromosomes, and restriction sites. Bulletin of the American Museum of Natural History. 2000;248: 1–200. doi: 10.1206/0003-0090(2000)248&lt;0001:POPBMC&gt;2.0.CO;2

[66] Boissinot S, Boursot P. Discordant Phylogeographic Patterns Between the *Y* Chromosome and Mitochondrial DNA in the House Mouse: Selection on the *Y* Chromosome? Genetics. 1997;146: 1019–1034. doi: 10.1093/genetics/146.3.1019 9215905 PMC1208032

[67] Metcalfe SE, O’Hara SL, Caballero M, Davies SJ. Records of Late Pleistocene–Holocene climatic change in Mexico—a review. Quaternary Science Reviews. 2000;19: 699–721. doi: 10.1016/S0277-3791(99)00022-0

[68] Jiménez-Barron O, García-Sandoval R, Magallón S, García-Mendoza A, Nieto-Sotelo J, Aguirre-Planter E, et al. Phylogeny, Diversification Rate, and Divergence Time of Agave sensu lato (Asparagaceae), a Group of Recent Origin in the Process of Diversification. Frontiers in Plant Sciences. 2020;11: 536135. doi: 10.3389/fpls.2020.536135 33240289 PMC7680843

[69] Good-Avila SV, Souza V, Gaut BS, Eguiarte LE. Timing and rate of speciation in Agave (Agavaceae). Proceedings of the National Academy of Sciences USA. 2006;103: 9124–9129. doi: 10.1073/pnas.0603312103 16757559 PMC1482577

[70] Van Devender T.R. The deep history of the Sonoran Desert. A Natural History of the Sonoran Desert. Tucson, Arizona: Oakland, California: Arizona-Sonora Desert Museum Press; University of California Press; 2015. pp. 61–69.

[71] McDonald J. A. Phytogeography and history of the alpine–subalpine flora of northeastern Mexico. Biological Diversity of Mexico: Origins and Distribution. New Yor, USA: Oxford Univ. Press; 1993. pp. 81–703.

[72] León-Paniagua L, Navarro-Sigüenza AG, Hernández-Baños BE, Morales JC. Diversification of the arboreal mice of the genus *Habromys* (Rodentia: Cricetidae: Neotominae) in the Mesoamerican highlands. Molecular Phylogenetics and Evolution. 2007;42: 653–664. doi: 10.1016/j.ympev.2006.08.019 17070711

[73] Ruiz EA, Rinehart JE, Hayes JL, Zuñiga G. Historical Demography and Phylogeography of a Specialist Bark Beetle, *Dendroctonus pseudotsugae* Hopkins (Curculionidae: Scolytinae). Environ Entomol. 2010;39: 1685–1697. doi: 10.1603/EN09339 22546468

[74] Bryson RW, Murphy RW, Graham MR, Lathrop A, Lazcano D. Ephemeral Pleistocene woodlands connect the dots for highland rattlesnakes of the *Crotalus intermedius* group: Diversification of montane rattlesnakes. Journal of Biogeography. 2011;38: 2299–2310. doi: 10.1111/j.1365-2699.2011.02565.x

[75] Hundertmark KJ, Shields GF, Udina IG, Bowyer RT, Danilkin AA, Schwartz CC. Mitochondrial Phylogeography of Moose (*Alces alces*): Late Pleistocene Divergence and Population Expansion. Molecular Phylogenetics and Evolution. 2002;22: 375–387. doi: 10.1006/mpev.2001.1058 11884162

[76] Hofreiter M, Stewart J. Ecological Change, Range Fluctuations and Population Dynamics during the Pleistocene. Current Biology. 2009;19: R584–R594. doi: 10.1016/j.cub.2009.06.030 19640497

[77] De Bruyn M, Hoelzel AR, Carvalho GR, Hofreiter M. Faunal histories from Holocene ancient DNA. Trends in Ecology & Evolution. 2011;26: 405–413. doi: 10.1016/j.tree.2011.03.021 21529992

[78] Scheinvar, Enrique. Filogeografía de *Agave lechuguilla* y patrones de distribución de Agave en México. CDMX, México: Universidad Nacional Autónoma de México; 2018.

[79] Valiente-Banuet A, Rojas-Martínez A, Arizmendi MDC, Dávila P. Pollination biology of two columnar cacti (*Neobuxbaumia mezcalaensis* and *Neobuxbaumia macrocephala*) in the Tehuacan Valley, central Mexico. American Journal of Botany. 1997;84: 452–455. doi: 10.2307/2446020

[80] Casas A, Valiente-Banuet A, Rojas-Marti´nez A, Davila P. Reproductive biology and the process of domestication of the columnar cactus *Stenocereus Stellatus* in Central Mexico. American Journal of Botany. 1999;86: 534–542. doi: 10.2307/2656814 10205073

[81] England, Angela E. Pollination Ecology of Agave palmeri in New Mexico, and Lanscape use of *Leptonycteris nivalis* in Relation to Agaves. PhD Dissertation. Albuquerque, New Mexico: The University of New Mexico; 2012.

[82] Festa Francesca, Ancillotto Leonardo, Santini Luca, Pacifici Michela, Rocha Ricardo, Toshkova Nia, et al. Bat responses to climate change: a systematic review. Biological Reviews. 2023;98: 19–33. doi: 10.1111/brv.12893 36054527 PMC10087939

[83] Ruiz Mondragon KY, Aguirre-Planter E, Gasca-Pineda J, Klimova A, Trejo-Salazar R-E, Reyes Guerra MA, et al. Conservation genomics of *Agave tequilana* Weber var. azul: low genetic differentiation and heterozygote excess in the tequila agave from Jalisco, Mexico. PeerJ. 2022;10: e14398. doi: 10.7717/peerj.14398 36415865 PMC9676017

[84] Laws James, Hill Michael T., Frey Jennifer K. Northernmost Record of the Long-nosed Bat (*Leptonycteris sp*.) in New Mexico: Conservation Implications. Western Wildlife. 2023;10: 6–10.

[85] Medellin Rodrigo A., Torres-Knoop Leonora. Evaluación de Riesgo de Extinción de Leptonycteris yerbabuenae de acuerdo al numeral 5.7 de la NOM‐059‐ SEMARNAT‐2010. MER *Leptonycteris yerbabuenae*. CONAMER; 2012. pp. 1–25.

[86] López-Hoffman L, Diffendorfer J, Wiederholt R, Bagstad KJ, Thogmartin WE, McCracken G, et al. Operationalizing the telecoupling framework for migratory species using the spatial subsidies approach to examine ecosystem services provided by Mexican free-tailed bats. E&S. 2017;22: art23. doi: 10.5751/ES-09589-220423

